# Adherence to the European Society of Cardiology (ESC) guidelines for chronic heart failure - A national survey of the cardiologists in Pakistan

**DOI:** 10.1186/1471-2261-11-68

**Published:** 2011-11-17

**Authors:** Sana Shoukat, Saqib A Gowani, Ather M Taqui, Rameez Ul Hassan, Zain A Bhutta, Anum I Malik, Sajjad A Sherjeel, Quratulanne Sheheryar, Sajid H Dhakam

**Affiliations:** 1 Internal Medicine, Emory University School of Medicine, Atlanta GA, USA; 2 Internal Medicine, University of Connecticut, Farmington CT, USA; 3 Neurology, Cleveland Clinic Foundation, Cleveland OH, USA; 4 Neurosurgery, Aga Khan University, Karachi, Pakistan; 5 Dow University of Health Sciences, Karachi, Pakistan; 6 Medical College, Aga Khan University, Karachi, Pakistan; 7 Khyber Medical College, Peshawar, Pakistan; 8 Section of Cardiology, Department of Medicine, Aga Khan University, Karachi, Pakistan

**Keywords:** Heart failure, cardiologist, guidelines, awareness

## Abstract

**Background:**

The aims of this study were to evaluate the awareness of and attitudes towards the 2005 European Society of Cardiology (ESC) guidelines for Heart Failure (HF) of the cardiologists in Pakistan and assess barriers to adherence to guidelines.

**Methods:**

A cross-sectional survey was conducted in person from March to July 2009 to all cardiologists practicing in 4 major cities in Pakistan (Karachi, Lahore, Quetta and Peshawar). A validated, semi-structured questionnaire assessing ESC 2005 Guidelines for HF was used to obtain information from cardiologists. It included questions about awareness and relevance of HF guidelines (See Additional File 1). Respondents' management choices were compared with those of an expert panel based on the guidelines for three fictitious patient cases. Cardiologists were also asked about major barriers to adherence to guidelines.

**Results:**

A total of 372 cardiologists were approached; 305 consented to participate (overall response rate, 82.0%). The survey showed a very high awareness of CHF guidelines; 97.4% aware of any guideline. About 13.8% considered ESC guidelines as relevant or very relevant for guiding treatment decisions while 92.8% chose AHA guidelines in relevance. 87.2% of respondents perceived that they adhered to the HF guidelines. For the patient cases, the proportions of respondents who made recommendations that completely matched those of the guidelines were 7% (Scenario 1), 0% (Scenario 2) and 20% (Scenario 3). Respondents considered patient compliance (59%) and cost/health economics (50%) as major barriers to guideline implementation.

**Conclusion:**

We found important self reported departures from recommended HF management guidelines among cardiologists of Pakistan.

## Background

The prevalence of heart failure (HF) continues to increase despite efforts at primary and secondary prevention[1]. Moreover, management of heart failure is complicated and requires extensive experience and knowledge of the current guidelines in effect. This can be tasking as they are updated frequently in accordance with new research trials and evolving recommendations.

Despite comprehensive guidelines being available, studies show failure of optimal management in patients[2-6]. Also, significant variation exists among hospitals in the implementation of published guidelines[7]. In real practice, not only is there inadequate prescription of recommended drugs, but also an issue with polypharmacy[4,8]. It is established that adherence to guidelines for heart failure management guidelines is a strong predictor of fewer hospitalizations and improved outcome in CHF patients[5,9-11].

Unlike the situation in developing countries, the South Asian subcontinent including Pakistan still faces an increase in mortality due to heart failure[12,13]. Pakistan is the 6^th ^most populous country in the world at 181 million. It is a developing country with 60% of the population living on less than US $2/day[14]. It faces the double burden of communicable as well as non-communicable illnesses including HF[15]. Healthcare provided at government hospitals is very poor. In most cases, people have to be able to pay for their medical expenses to receive optimum health care. Hence, the health system is itself not strong enough to handle the increasing costs associated with decompensated heart failure hospitalizations. Studies in Pakistan have demonstrated a shortfall in management of cardiovascular disorders [16-18] In order to derive effective measures to improve chronic heart failure management skills among the cardiologists in Pakistan, it is imperative to assess their current knowledge and awareness of existing guidelines. At present, no national guidelines exist in Pakistan; cardiologists manage patients in accordance with the AHA or ESC guidelines.

The aim of this study was to evaluate the awareness of and attitudes of cardiologists in Pakistan towards the latest ESC guidelines for CHF and assess barriers to adherence to guidelines.

## Methods

### Study Design

This cross sectional study was conducted among practicing cardiologists in the capital cities of the four provinces of Pakistan: Karachi, Lahore, Quetta and Peshawar in the provinces of Sindh, Punjab, Balochistan and North Western Frontier Province, respectively. The study was conducted from March to July 2009.

The study was conducted in compliance with the Declaration of Helsinki and the Department of Medicine Ethics committee approved the research protocol. Informed consent has been obtained from the participants.

### Participants

Cardiologist was defined as a physician whose practice included >80% patients with cardiac diseases, regardless of post graduate training. We have used the same definition in our previous paper by Gowani et al[16]. The reason behind this is the unregulated medical practice in the country leading to considerable heterogeneity among physicians practicing cardiology in Pakistan. Hence, this definition allowed us to capture all practitioners providing specialty cardiac care in the field, regardless of credentials.

A list of cardiologists in each city was prepared by reviewing the membership directory maintained by the Pakistan Cardiac Society. In addition to that marketing divisions of major pharmaceutical companies doing business in Pakistan were also contacted to add and reinforce our list of practicing cardiologists. Furthermore, in each city, we contacted major hospitals (defined as housing > 100 beds) and procured the names of the physicians listed as staff cardiologists. Using this multi pronged approach, a list of cardiologists was developed for each of the 4 cities.

All cardiologists on our comprehensive list were contacted and invited to participate in the survey. All participants signed an informed-consent form. Full confidentiality was assured and stating the participants' name on the questionnaire was optional. Cardiologists practicing at the investigators' university hospital (Aga Khan University, Karachi, Pakistan) were excluded from the study. Cardiologists were approached personally by interviewers (volunteers) in their offices. If the respondent was busy, a later time was obtained. All questionnaires were filled by respondents while interviewers present. 80% of the respondents filled the questionnaire on the first visit. There was no significant difference in awareness of guidelines between those who responded immediately and the ones on subsequent visits.

### Study Instrument

The questionnaire was adopted from Leif Erhardt et al with the authors permission[19]. The questionnaire was developed in English language. Pretesting was carried out among the cardiologists of the study institution. These were not approached during data collection. The pretest sample comprised of 12 respondents. A few amendments were made to clarify ambiguities in a few parts of the questionnaire. Trained medical officers then approached individual cardiologists at their offices, in person, and invited them to participate. Because all medical education in Pakistan is conducted in English, we felt confident that there would be no comprehension issues with an English language questionnaire. However, the medical officers were available to answer any inquiries.

The original questionnaire was developed by an expert panel of European Cardiologists, including two who were involved in the development and publication of the 2005 ESC guidelines. The expert panel developed three fictitious patient case scenarios that were designed to reflect different aspects of the management of patients with CHF. In summary, they were: a 69-year-old man with a history of hypertension and myocardial infarction, newly diagnosed with heart failure; a 70-year-old woman diagnosed with heart failure 2 years ago, now with exacerbation of symptoms; and a 75- year-old woman with CHF and preserved left ventricular function. A selection of treatment options, including some that were not consistent with the 2005 ESC guidelines, was provided. This was adopted from a survey conducted by Erhardt et al among European Cardiologists with the permission of their authors[19].

### Definitions

*Non invasive cardiologist *was defined as a practitioner who does not perform invasive procedures, such as coronary angiography or percutaneous coronary interventions (PCI). Invasive non-interventional cardiologist was defined as someone who performs invasive diagnostic coronary procedures, such as left and right heart catheterization and coronary angiography but no interventions. Interventional cardiologist was defined as someone who performs PCI or peripheral vascular intervention, in addition to invasive diagnostic procedures.

*Teaching hospital *was defined as a university- or medical school- affiliated hospital, a hospital that supports a residency or fellowship program accredited by Pakistan's College of Physicians and Surgeons (the country's regulatory body for postgraduate medical education), or a hospital that met both criteria.

*Cardiologist *were said to have a bona fide cardiology qualification if their academic degree required formal structured cardiology training (e.g., Fellow of the College of Physician and Surgeons [Pakistan] in cardiology, member of Royal College of Physicians, American Board of Internal Medicine subspecialty certification in cardiovascular diseases).

*Professional meeting *was defined as a major professional society-initiated meeting in the field of cardiology held anywhere in the world, including Pakistan.

### Data Analysis

The data was entered and analyzed in Statistical Package for Social Sciences 17.0 (SPSS, Inc., Chicago, IL, USA). Descriptive statistics were performed for physician demographics and responses to questions on awareness of guidelines. The results were recorded as frequencies, means ± standard deviations (SD) and p-values. Tables and figures were used for comprehensive viewing of the results.

## Results

A total of 372 cardiologists were approached; 305 consented to participate (overall response rate, 82.0%; 85.6% for Karachi, 81.3% for Lahore, 71.4% for Quetta, 75.5% for Peshawar). Three hundred and five cardiologists from four major cities completed the survey: Karachi (n = 167), Lahore (n = 78), Peshawar (n = 40), Quetta (n = 20). Table 1 shows their background and practice details. The mean age of the cardiologists was 42.1 ± 9.7 years and 89.2% were males. The mean time period spent treating heart failure was 12.3 ± 8.6 years. 68.2% of the cardiologists had completed their postgraduate training in Pakistan, followed by 26.6% in the UK or Europe. The majority of cardiologists (82.6%) saw both inpatients and outpatients in their practice. The average number of inpatients and outpatients seen per week was 23.5 ± 38.7 and 23.4 ± 14.9 respectively.

**Table 1:** Background and practice details of the cardiologists.

	n (%) or Mean ± SD
**Age (years)**	42.1 ± 9.7

**Males**	272 (89.2)

**Period of cardiology practice (years)**	12.4 ± 8.6

**Period of treating heart failure patients (years)**	12.3 ± 8.6

**Highest qualification**	
MBBS or equivalent basic medical degree	18 (5.9)
FCPS medicine	62 (20.3)
FCPS cardiology	52 (17.0)
MRCP	62 (20.3)
American Board of IM	5 (1.6)
American Board of IM, Subspecialty of Cardiology	5 (1.6)
Fellowship	18 (5.9)
Diploma in Cardiology	82 (26.9)

**Completion of post-graduate cardiology training**	
Pakistan	208 (68.2)
USA	13 (4.3)
UK or Europe	81 (26.6)

**Nature of practice**	
Only out-patient	30 (9.8)
Only inpatient	23 (7.5)
Both	252 (82.6)

**Type of cardiology practice**	
Non-invasive cardiologist	182 (59.7)
Interventional cardiologist	89 (29.2)
Invasive non-interventional cardiologist	34 (11.1)

**Affiliation with teaching hospital**	196 (64.3)
As full-time faculty	159 (81.1)
As part-time faculty	37 (18.9)

**Average number of patients seen in**	
Outpatient	23.4 ± 14.9
Inpatient	23.5 ± 38.7

MBBS, Bachelor of Medicine Bachelor of Surgery; FCPS, Fellow of college of Physicians and Surgeons; MRCP, Membership of the Royal Colleges of Physicians; IM, Internal Medicine

Table 2 shows the responses of the cardiologists to questions on awareness of the guidelines. 92.1% reported awareness of AHA guidelines, 40.7% of ESC and only 11.8% of Pakistan guidelines. Almost all (99.0%) of respondents believed that the CHF guidelines were relevant or very relevant in influencing treatment choices. In prompted questioning, the AHA guidelines turned out to be the most relevant (92.8%). The majority (87.2%) of respondents reported that they closely follow the guidelines in general. Also 75.7% of the cardiologists reported that they were well informed with the latest update of the guidelines they follow. The major sources of information about CHF guidelines were reported to be journals (80.0%) followed by internet (72.8) and post-graduate training (63.9%).

**Table 2:** Responses to questions on awareness of guidelines

	n (%)
**Awareness of existing guidelines**	
Not aware of any	8 (2.6)
American (AHA)	281 (92.1)
European (ESC)	124 (40.7)
Pakistan	36 (11.8)

**Relevance of CHF guidelines in making treatment choices**	
Not relevant	3 (1.0)
Relevant	165 (54.1)
Very relevant	137 (44.9)

**Most relevant guidelines**	
AHA	283 (92.8)
ESC	42 (13.8)
Pakistan	19 (6.2)

**Do you follow guidelines you are aware of closely?**	
Yes	266 (87.2)

**Are you well informed with the latest update of the guidelines you follow?**	
Yes	231 (75.7)

**Major source of information about CHF guidelines**	
My training	195 (63.9)
Colleagues	70 (23.0)
Journals	244 (80.0)
Professional meetings	180 (59.0)
Drug representatives	19 (6.2)
Internet	222 (72.8)

**Journals read on a regular basis**	
Heart	168 (55.1)
NEJM	120 (39.3)
Circulation	182 (59.7)
Lancet	83 (27.2)
JPMA (Local indexed journal)	89 (29.2)

AHA, American Heart Association; ESC, European Society of Cardiology; JPMA, Journal of Pakistan Medical Association

Table 3 shows the association of relevant variables with awareness of AHA and ESC guidelines. Cardiologists who possessed a higher level of qualifications reported significantly higher awareness of AHA guidelines than those with relatively low qualifications (Diploma in Cardiology and MBBS or equivalent degree): 96.6% vs. 83%, p value <0.0001. A similar pattern was noted for ESC guidelines but the difference was not significant: 44.4% vs. 33%, p value = 0.057. Cardiologists who had spent more than five years in heart failure practice reported significantly higher awareness of ESC guidelines: 44% vs. 32.2%, p value = 0.046. A significant difference was not noted for awareness of AHA guidelines; the awareness was high regardless of the number of years spent in heart failure practice. The nature of practice did not appear to influence awareness of either of the guidelines. Cardiologists who had completed their post-graduate training abroad reported significantly higher awareness of ESC guidelines than those who completed training in Pakistan: 56.7% vs. 33.2% (p <0.0001).

**Table 3:** Association of relevant variables with awareness of AHA and ESC guidelines on CHF

	Awareness of AHA guidelines n (%)	P value	Awareness of ESC guidelines n (%)	P value
**Qualification**				
Low	83 (83.0)	< 0.0001	33 (33.0)	0.057
High	198 (96.6)		91 (44.4)	

**Period of heart failure practice**				
≤ 5 years	71 (88.8)	0.191	25 (31.2)	0.046
> 5 years	210 (93.3)		99 (44.0)	

**Nature of practice**				
Outpatient only	26 (86.7)	0.086	7 (23.3)	0.059
Inpatient only	19 (82.6)		7 (30.4)	
Both	236 (93.7)		110 (43.7)	

**Post-graduate training**				
Pakistan	191 (91.8)	0.773	69 (33.2)	< 0.0001
Other	90 (92.8)		55 (56.7)	

Low qualification includes Diploma in Cardiology and MBBS or equivalent degree. High includes the rest.

P value calculated using chi-square test.

**Figure 1:**
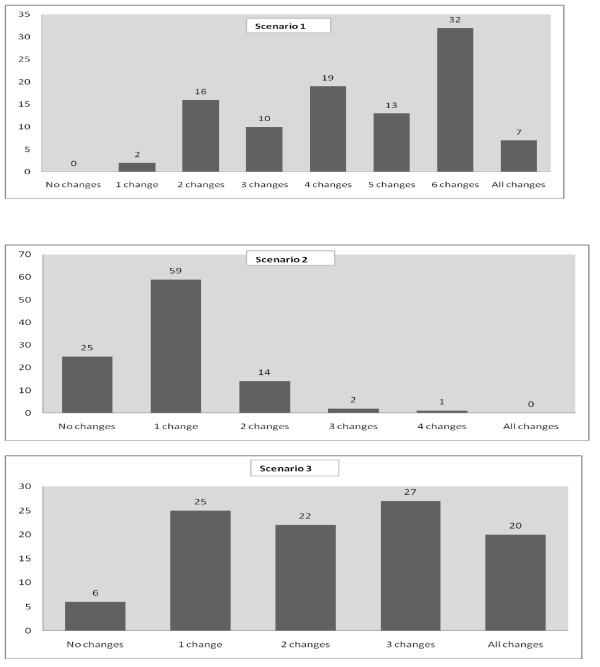
**Summary of responses for the three patient scenarios**. Respondents (%) with treatment changes matching those of the panel. Scenario 1: Patient with newly-diagnosed heart failure; Scenario 2: Patient with uncontrolled CHF; Scenario 3: Patient with CHF and preserved left ventricular function.

Figure 1 shows the responses of the cardiologists to management options for the three patient scenarios (Please see questionnaire for scenarios in appendix). Overall, the cardiologists' management recommendations concurred with the guidelines. However, respondents did more poorly on Scenario 2 (patient presenting with uncontrolled CHF) compared to the other two scenarios: 84% made only one or zero correct treatment recommendations (out of 5 correct choices) compared to 2% and 31% in Scenario 1 and 3 respectively. The proportions of respondents who made recommendations that completely matched those of the guidelines were 7% (Scenario 1), 0% (Scenario 2) and 20% (Scenario 3). Cardiologists made wrong choices with the type of therapy and the appropriate doses.

**Figure 2:**
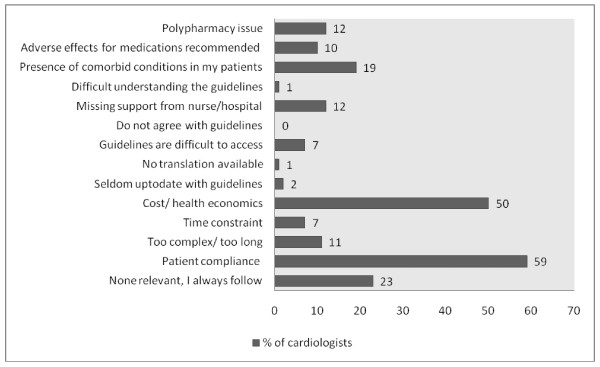
**Cardiologists' perception of barriers for adherence to guidelines for CHF**.

**Figure 3:**
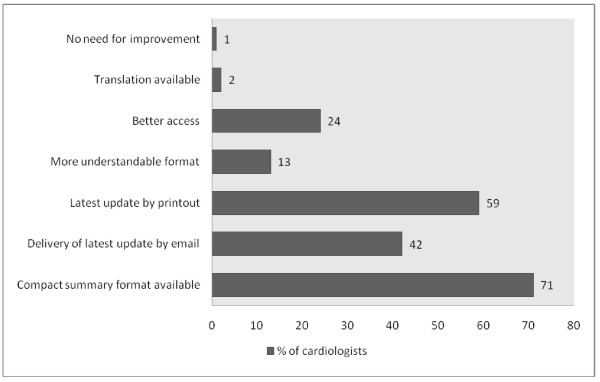
**Possible strategies to improve implementation of guidelines**.

Figure 2 illustrates the perceived barriers for adherence to guidelines. The top three barriers identified were patient compliance (59%), cost/health economics (50%) and comorbid conditions of patients (19%). The cardiologists were also asked about the possible strategies to improve adherence to guidelines; the top three strategies identified were availability of a compact summary format (71%), latest update by printout (59%) and delivery of latest update by email (42%). This is shown in Figure 3.

Responses were compared across the four cities. There was a significant difference in responses to scenario 3, with cardiologists from Karachi having a greater number of correct responses (making 3 or all correct responses) than their peers in the other three cities (67% vs. 16.7-30.0%, p < 0.0001). When comparing responses across cities to top three barriers to following guidelines, the only significant difference was observed in the third barrier: presence of comorbid conditions in patients. When comparing responses across cities to top three strategies to improve adherence, significant differences were observed in two strategies: compact summary available and latest update available by printout. Cardiologists from Karachi opted for these two strategies more frequently than their peers.

## Discussion

This national survey highlights that the majority of practicing cardiologists of Pakistan are aware of existence of guidelines for heart failure and consider them relevant for heart failure management of their patients. However, when their grasp of the ESC guidelines was assessed by patient scenarios, only a very few could make the management choices exactly in accordance with the ESC guidelines based on an expert panel used by Erhardt et al[19].

Most common cause of heart failure is coronary artery disease in both Europe and South Asians. However, south Asians have a greater prevalence of premature heart disease given heavy burden of diabetes and other risk factors[20]. This necessitates aggressive risk factor control in Pakistani population that is tailored to fit the population behavior and health care system of Pakistan. However, there is no validated guideline that providers can follow. Most physicians are trained via a European system and are familiar with the European guidelines. It does remain a question whether adherence to these guidelines will be adequate to improve HF mortality in Pakistan.

Our results indicate that despite high awareness of importance of managing patients according to guidelines, the knowledge was either scarce or that physicians were unable to put that knowledge into practice. The most frequently reported barriers to use of these guidelines were lack of patient compliance and cost/health economics. Most cardiologists felt that availability of a more compact version of the guidelines would be effective in increasing adherence to them.

Most cardiologists in Pakistan have undergone post graduate training in Pakistan or the United Kingdom (UK)/Europe. Postgraduate medical training within Pakistan resembles the UK training. We expected the practicing physicians to be more aware of European.. However, our results indicate AHA being more popular among Pakistani physicians than the ESC guidelines. We presume that this is inconsequential as the ESC and AHA guidelines are comparable in recommendations[21].

The lack of adequate adherence to recommended guidelines has been reported in several studies across the globe. The Get With the Guidelines -- Heart Failure Program, a US based study conducted by Patel et al. showed patients with increased severity of renal dysfunction and HF were less likely to receive important guideline-recommended therapies despite higher mortality rates[22]. IMPROVE HF was another US based, prospective cohort study designed to characterize the management of patients with chronic HF and left ventricular ejection fraction ≤35% in outpatient cardiology practices. They found that the adherence for recommended adjunctive therapies was extremely low[23]. Similarly, the EuroHeart Failure Survey program showed that the prescription of recommended medications including ACE inhibitors and beta-blockers was limited and that the daily dose was lower than recommended[4]. Rywik et al also pointed out the concern of underuse of beta blockers and use of Calcium channel blockers in HF patients in Poland, Europe[24].

We grouped MBBS and Cardiology diplomas as low level of qualification and found that these physicians tended to be significantly less aware of the existence of AHA guidelines. This trend was true for the ESC guidelines too but did not reach significance. This is concerning as almost a third of the listed cardiologists of the country were merely MBBS or Diploma in Cardiology and not trained well enough to follow latest evidence published. It is imperative that some serious effort is made to update these health care providers.

Very few physicians made all the choices as recommended by the ESC guideline expert panel. Compared to Erhardt et al., our cardiologists did very poorly for scenario 2 - a 70 year old female patient with HF and with acute exacerbation of symptoms[19]. Perhaps adherence to guidelines is not only dependent on physician characteristics but also patient profiles. The Improve HF study by Yancy et al. showed that older patients, particularly older women, were significantly less likely to receive guideline-indicated HF therapies[25]. Kasje et al also showed that guideline recommended use of ACE Inhibitors was more linked to patient characteristics[26].

Our respondents did best on scenario 3 - a female patient with CHF and preserved left ventricular function showing that physicians were more comfortable in applying guideline recommendations in a stable patient than in a patient with exacerbation. This finding can be used to focus more on physician education on guideline recommendations for hospitalized patients with exacerbations.

### Barriers to implementation of guidelines

The cardiologists of Pakistan cited patient compliance and cost/health economics as the most common barriers to implementation of guidelines. This is in contrast to Erhardt et al where guideline complexity was seen as an important barrier among respondents[19]. Barriers are dependent on the existing health system. Pakistan is a developing country with poor health indicators[14]. Moreover, government provided health insurance is nonexistent. To attain quality health care, the patient has to bear the cost himself. It becomes inevitable that physicians are forced to recommend suboptimal therapy to patients with financial constraints. Sturm et al. showed that prescribed drug volume and choice of drug regimes for HF were country specific [27]. Therefore, health economics is one crucial factor within the Pakistani health-care system affecting guideline implementation.

Literature also suggests that patient factors, including compliance, socioeconomic factors or demographics are important in establishing guideline adherence among physicians[25,26]. A survey conducted by Keefe et al showed that most physicians did not follow guideline recommendations because the suggestions were felt to be inapplicable to their patients or unlikely to be tolerated [28]. In another study, patient age >75 years remained an independent predictor of under-prescription of CHF drugs [8]. Our physicians frequently reported patient compliance as a barrier demonstrating that patient factors are most relevant when assessing guideline adherence.

### Improving adherence to guidelines

Most physicians believed that a more compact version, latest update by printout and via email will help them in implementing guidelines better in their practices. Some interventions have been tried out previously including standard education on guideline adherence (GA) in general practice and new, multifaceted intervention (educational train-the-trainer course plus pharmacotherapy feedback = TTT)[29,30]. It was seen that the TTT intervention did not prove any more beneficial than conventional education of physicians. Nevertheless, it is important to take into account what physicians believe themselves as possible ways of learning more about guidelines. The Pakistan Cardiac Society (PSC) can make a summarized version available derived from AHA/ESC and then send it to all cardiologists via printouts and emails. Later updates can be sent the same way too. For improving adherence, we also recommend that PCS provide a list of trade names for drugs which are cheaper so that it is easier for physicians to overcome the cost barrier.

### Limitations

The main strength of the study was its high overall response rate (82.0%) but there were also a number of limitations. First, since all practicing cardiologists were not eligible to participate, this may have introduced selection bias through the sampling method. However, we believe that our sample was relatively representative of the country's cardiologists as participants were selected from 4 major cities across Pakistan. Second, Peshawar and Quetta were underrepresented in our study due to their high refusal rate to participate. Third, although we made sure to include every cardiologist practicing in the respective city, there still is a possibility that we may have missed a few. Fourth, this study only assessed the performance of the cardiologists in major cities. We cannot extrapolate these data to those practicing in rural areas. That said, in Pakistan, few cardiologists practice in rural areas, and patient from these areas have to visit major cities to access tertiary care facilities. Fifth, the majority of the participants were men. This was related to the paucity of the female cardiologists in Pakistan. Additionally, results are largely dependent on the definition of cardiologist. Finally, it should be noted that this was a survey of physicians regarding their practices; as a result, the findings were more subjective than they would have been in a study that used an objective measure of performance, such as chart review. Because physicians may overestimate their performance relative to treatment guidelines, our results may underestimate cardiologists' divergence from established practices.

## Conclusions

Based on our review of the indexed medical literature, this is the first study to examine awareness of and adherence to HF guidelines among cardiologists in Pakistan. The survey results indicate deviations from recommended evidence-based practice, which may lead to suboptimal treatment and patient outcomes. Cost/Health economics is a major barrier to guideline adherence specific to Pakistani cardiologists.

## Conflict of interests

The authors have indicated that they have no conflicts of interests regarding the content of this article.

## Authors' contributions

SS and SAG conceptualized the study and were involved in the study design. RUH, ZAB, AIM, SAS and QS collected the data. AMT, SAG and SS were involved in the data analysis and data interpretation. SS, SAG and AMT prepared the manuscript. SHD provided critical feedback and guidance and was responsible for the study's ongoing management. All authors read and approved the final manuscript.

## Pre-publication history

The pre-publication history for this paper can be accessed here:

http://www.biomedcentral.com/1471-2261/11/68/prepub

## Supplementary Material

Additional file 1 **Questionnaire**. Description: Questionnaire that was administered to participants. Click here for file
