# Supplementary material for: Adherence to the European Society of Cardiology (ESC) guidelines for chronic heart failure - A national survey of the cardiologists in Pakistan
Source: BMC Cardiovasc Disord. 2011 Nov 17;11:68. doi: 10.1186/1471-2261-11-68 (PMC3250933; doi:10.1186/1471-2261-11-68)
Supplement: Additional file 1 — Questionnaire. Description: Questionnaire that was administered to participants. [file 1471-2261-11-68-S1.DOC]

**Heart failure Questionnaire**

1. Gender

Male

Female

2. Age (in years)? __________

3. Year of graduation from medical school _________________

3. How long (years) have you been practicing cardiology? _________________

4. How long (years) have you been treating patients with CHF? _____________________

5. What is your **highest** qualification?

M.B.B.S or equivalent basic medical degree

FCPS medicine

FCPS cardiology

MRCP

American Board of Internal Medicine

American Board of Internal Medicine, Subspecialty of Cardiology

Fellowship

Diploma in Cardiology

Other (Please specify)_______________

6. Where did you complete your **post-graduate** cardiology training?

Pakistan

U.S.A.

U.K. or Europe

Other country……………………………….

7. How would you classify your practice?

I see out-patients only

I see in-patients only

I see both out and in patients

8. What sort of cardiologist would you say you are?

Non-invasive cardiologist

Interventional cardiologist (I do coronary interventions)

Invasive non-interventional cardiologist (I do diagnostic cath but not interventions)

9. Are you affiliated with a teaching hospital?

No

Yes If so, name: _______________________________

Full time faculty  Part time faculty

10. Approximately how many patients do you see in an average day?

In the outpatient _____________

In the hospital _____________

| **Scenario 1**  69-year-old man with a history of hypertension for 10 years and myocardial infarction 1 year ago presenting with dyspnoea; chest pain 1 week ago but not currently experiencing pain; no previous HF.   - Chest X-ray − congestion - Rapid atrial fibrillation – HR 125 bpm - ECG − Q waves over the anterior wall (leads V1-V5) - Echocardiogram   • Reduced ejection fraction (35%)  • Anterior wall shows reduced contractions   - Lipids   Total cholesterol 5.4mmol/L (210mg/dL)  LDL 3.6mmol/L (140mg/dL)  HDL 0.9mmol/L (35 mg/dL)   - Enzymes negative - Physical examination and other lab tests normal - BP 155/85 mm Hg - Medication   • 50mg atenolol - started 10 years ago  • 5mg enalapril - added 1 year ago  Medication unchanged over the last year Treatment options 1. Enalapril  No change  Discontinue  Increase dose  Decrease dose  Switch to different product of the same class  2. Atenolol  No change  Discontinue  Increase dose  Decrease dose  Switch to  bisoprolol  carvedilol  metoprolol succinate  nebivolol  other beta-blocker  3. Which therapies would you suggest in addition to or instead of as 1st line treatment, if any at all? And 2nd line (if patient remained symptomatic or did not tolerate treatment)?   |  | 1^st^ line | 2^nd^ line | | --- | --- | --- | | Intravenous (IV) loop diuretics |  |  | | other diuretics |  |  | | Aldosterone antagonists |  |  | | Angiotensin II receptor blockers (ARBs) |  |  | | Cardiac glycosides |  |  | | Calcium antagonists |  |  | | Statins |  |  | | Nitrates |  |  | | Acetyl-salicylic acid (ASA) |  |  | | other Thrombocyte aggregation inhibitors |  |  | | Warfarin |  |  | | other anticoagulants |  |  | | A different product class not mentioned above |  |  | | would not add any therapy |  |  | |
| --- | --- | --- | --- | --- | --- | --- | --- | --- | --- | --- | --- | --- | --- | --- | --- | --- | --- | --- | --- | --- | --- | --- | --- | --- | --- | --- | --- | --- | --- | --- | --- | --- | --- | --- | --- | --- | --- | --- | --- | --- | --- | --- | --- | --- | --- |

| **Scenario 2**  70-year-old woman with HF for 2 years experiences a deterioration in condition presenting with increased dyspnoea, tiredness and dizziness.     - BP 170/70 mm Hg, HR 72 bpm - Chest X-ray − chronic congestion with Kerley’s B-lines and pleural fluid 3 cm bilaterally - Echocardiogram: Severely reduced ejection fraction (20-25%) - Serum creatinine − 195mmol/L (2.2mg/dL) - Sodium 136 and potassium 3.9 - Functionally NYHA class IIIB - Medication:   • 5mg enalapril – started 2 years ago  • 40mg bid (twice daily) frusemide / furosemide – started 2 years ago  • 50mg metoprolol succinate – added 6 months ago    Thereafter stable medication.  Treatment options  1. Enalapril  No change  Discontinue  Increase dose  Decrease dose  Switch to different product of the same class  2. Metoprolol succinate  No change  Discontinue  Increase dose  Decrease dose  Switch to  bisoprolol  carvedilol  atenolol  nebivolol  other beta-blocker  3. Frusemide  Furosemide oral  No change  Discontinue  Increase dose  Decrease dose  Switch to other oral diuretics  4. Which therapies would you suggest in addition to or instead of as 1st line treatment, if any at all? And 2nd line (if patient remained symptomatic or did not tolerate treatment)?     |  | 1^st^ line | 2^nd^ line | | --- | --- | --- | | Intravenous (IV) loop diuretics |  |  | | Aldosterone antagonists |  |  | | Angiotensin II receptor blockers (ARBs) |  |  | | Cardiac glycosides |  |  | | Calcium antagonists |  |  | | Statins |  |  | | Nitrates |  |  | | Acetyl-salicylic acid (ASA) |  |  | | other Thrombocyte aggregation inhibitors |  |  | | Warfarin |  |  | | other anticoagulants |  |  | | A different product class not mentioned above |  |  | | would not add any therapy |  |  | |
| --- | --- | --- | --- | --- | --- | --- | --- | --- | --- | --- | --- | --- | --- | --- | --- | --- | --- | --- | --- | --- | --- | --- | --- | --- | --- | --- | --- | --- | --- | --- | --- | --- | --- | --- | --- | --- | --- | --- | --- | --- | --- | --- |

| **Scenario 3.**  75-year-old woman, treated for hypertension for the last 15 years presents with acute onset of increasing dyspnoea at rest.   - Patient in stable condition (i.e. in sinus rhythm) - Chest X-ray − pulmonary oedema - ECG − signs of left ventricular hypertrophy (LVH), HR 92 bpm - Echocardiogram   • Clear signs of left ventricular hypertrophy  • Normal ejection fraction (~50%)  • Signs of decreased relaxation indicating stiffness of the LV wall   - BP 190/90 mm Hg - No history of chest pain - All other organs normal - No history of coronary heart disease or diabetes - Electrolytes and kidney function normal - Medication   • 12.5mg hydrochlorothiazide – started 15 years ago  • 80mg bid (twice daily) verapamil – added 1year ago since her systolic pressure was not adequately controlled.  Not seen by her doctor for the last 6 months Treatment options  1. Verapamil   No change  Discontinue  Increase dose  Decrease dose  Switch to different product of the same class  2. Hydrochlorothiazide  No change  Discontinue  Increase dose  Decrease dose  Switch to other oral diuretics  3. Which therapies would you suggest in addition to or instead of as 1st line treatment, if any at all? And 2nd line (if patient remained symptomatic or did not tolerate treatment)?   |  | 1^st^ line | 2^nd^ line | | --- | --- | --- | | Intravenous (IV) loop diuretics |  |  | | Beta-Blockers |  |  | | ACEis |  |  | | Aldosterone antagonists |  |  | | Angiotensin II receptor blockers (ARBs) |  |  | | Cardiac glycosides |  |  | | Statins |  |  | | Nitrates |  |  | | Acetyl-salicylic acid (ASA) |  |  | | other Thrombocyte aggregation inhibitors |  |  | | Warfarin |  |  | | other anticoagulants |  |  | | A different product class not mentioned above |  |  | | would not add any therapy |  |  | |
| --- | --- | --- | --- | --- | --- | --- | --- | --- | --- | --- | --- | --- | --- | --- | --- | --- | --- | --- | --- | --- | --- | --- | --- | --- | --- | --- | --- | --- | --- | --- | --- | --- | --- | --- | --- | --- | --- | --- | --- | --- | --- | --- | --- | --- | --- |

11. Are you aware of any guidelines existing for management of congestive heart failure?

*(Tick as many as applicable)*

Not aware of any

American (AHA)

European (ESC)

Pakistan

12. In your opinion, for guiding their treatment choices, you would rate these CHF guidelines as.

Not relevant

Relevant

Very relevant

13. Which of these guidelines do you find most relevant for management of your CHF patients?

*(Tick as many as applicable)*

American (AHA)

European (ESC)

Pakistan

Other: ___________________________

14. Do you follow CHF guidelines you are aware of in general closely?

Yes

No

15. Are you well informed with the latest update of the guidelines you follow?

Yes

No

16. If yes to Q15, what is your source for the latest updates?

My training during medical school, residency and fellowship

Colleagues

Journals

Professional meetings

Drug Reps

Internet

17. In your opinion, what are the barriers that keep you from following the guidelines closely in your management of CHF patients?

*(Tick as many as applicable)*

None relevant, I always follow

Patient compliance (my patients may drop out for the recommended drug regimens in guidelines)

Too complex/ too long

Time constraint

Cost/ health economics

I am seldom uptodate with the guidelines

There’s no translation available

Guidelines are difficult to access

Do not agree with guidelines

Missing support from nurse/hospital

Difficult understanding the guidelines

Presence of comorbid conditions in my patients

Adverse effects or contraindications for medications recommended by guidelines

polypharmacy issue

18. What would you regard as the major source of information about CHF guidelines for your practice?

*(Tick as many as applicable)*

My training during medical school, residency and fellowship

Colleagues

Journals

Professional meetings

Drug Reps

Internet

19. Which of the following journals are you subscribed to or read on a regular basis?

*(Tick as many as applicable)*

Heart

NEJM

Circulation

Lancet

JPMA

Other………………………..

None

20. How often do you attend Cardiology meetings and scientific conferences?

Once in every 3 months

Once in every 6 months

Once every year

Once in a few years

Never been to one
